# Usnic acid and tannic acid as inhibitors of coccidia and *Clostridium perfringens*: alleviating necrotic enteritis and improving intestinal health in broiler chickens

**DOI:** 10.1186/s40104-025-01201-0

**Published:** 2025-05-12

**Authors:** Huiping Xu, Minghao Yang, Jianyang Fu, Huiyuan Lv, Jiang Guo, Changji Lu, Zengpeng Lv, Yuming Guo

**Affiliations:** 1 State Key Laboratory of Animal Nutrition and Feeding, College of Animal Science and Technology, China Agricultural University, Beijing, 100193 China; 2 Beijing Centre Biology Co., Ltd., Beijing, 100193 China; 3 Fujian Sunner Development Co., Ltd., Nanping, 354199 China

**Keywords:** Broiler chickens, Intestinal health, Necrotic enteritis, Tannic acid, Usnic acid

## Abstract

**Background:**

Necrotic enteritis (NE) in broiler chickens leads to significant economic losses in poultry production. This study examined the inhibitory effects of usnic acid and tannic acid on coccidia, sporozoite, and *Clostridium perfringens* and assessed their influence on growth performance and intestinal health in NE-challenged broilers through in vitro and in vivo experiments.

**Methods:**

The in vitro experiment included 5 treatment groups: the negative control (NC), 2 μmol/L diclazuril (DZ), 30 μmol/L usnic acid (UA), 90 μmol/L tannic acid (TA), and 15 μmol/L usnic acid + 45 μmol/L tannic acid (UTA) groups. The in vivo experiment involved 320 broilers divided into four groups: PC (NE-challenged), SA (500 mg/kg salinomycin premix + NE-challenged), UA (300 mg/kg usnic acid + NE-challenged), and UTA (300 mg/kg usnic acid + 500 mg/kg tannic acid + NE-challenged) groups.

**Results:**

In the in vitro study, the UA, TA, and UTA treatments significantly increased apoptosis in coccidian oocysts and sporozoites, lowered the mitochondrial membrane potential (*P* < 0.05), and disrupted the oocyst structure compared with those in the NC group. UA and TA had inhibitory effects on *C. perfringens*, with the strongest inhibition observed in the UTA group. The in vivo results demonstrated that the SA group presented significantly improved growth performance on d 13, 21, and 28 (*P* < 0.05), whereas the UA and UTA groups presented improvements on d 13 and 21 (*P* < 0.05). The SA, UA, and UTA treatments reduced the intestinal lesion scores by d 28 and the fecal coccidian oocyst counts from d 19 to 21 (*P* < 0.05). Compared with the PC group, the UA and UTA groups presented lower intestinal sIgA levels and CD8^+^ cell percentages (*P* < 0.05), with a trend toward a reduced CD3^+^ cell percentage (*P* = 0.069). The SA, UA, and UTA treatments significantly reduced the serum diamine oxidase activity, crypt depth, and platelet-derived growth factor levels in the intestinal mucosa while increasing the villus height to crypt depth ratio and number of goblet cells (*P* < 0.05). The UTA treatment also significantly increased the acetate and butyrate concentrations in the cecum (*P* < 0.05). With respect to the gut microbiota, significant changes in β diversity in the ileum and cecum were observed in the SA, UA, and UTA groups, indicating that the microbial community compositions differed among the groups. *Romboutsia* dominated the SA group, Bacillales dominated the UA group, and Lactobacillales and Lachnospirales dominated the UTA group in the ileal microbiota. In the cecal microbiota, *Lactobacillus*, *Butyricicoccus*, and *Blautia* abundances were significantly elevated in the UTA group (*P* < 0.05).

**Conclusion:**

Usnic acid and tannic acid induce apoptosis in coccidia and sporozoites by lowering the mitochondrial membrane potential. Both usnic acid alone and in combination with tannic acid alleviate NE-induced adverse effects in broilers by modulating intestinal immunity, altering the microbial composition, and improving intestinal barrier function. Compared with usnic acid alone, the combination of usnic acid and tannic acid had superior effects, providing a promising basis for the development of effective feed additive combinations.

## Background

Necrotic enteritis (NE) is a common intestinal disease that causes considerable economic losses in the poultry sector, with global losses exceeding $2 billion annually [1]. The primary etiological agent of NE is *Clostridium perfringens*, an anaerobic Gram-positive bacterium commonly present in soil, sewage, and the gastrointestinal tracts of animals and humans. Several factors predispose poultry to NE, including co-infection with coccidia and diets rich in non-starch polysaccharides. Coccidial infections damage the intestinal mucosa, causing plasma protein leakage into the intestinal lumen and promoting mucus secretion, which creates favorable conditions for *C. perfringens* proliferation, culminating in NE development [2, 3]. Diclazuril is a commonly used anticoccidial drug that is primarily used to control coccidiosis in poultry and other animals. It helps reduce intestinal damage caused by coccidia by inhibiting their reproduction, thereby alleviating the development of NE induced by coccidia.

Antibiotics are widely employed in animal agriculture to increase growth and prevent disease; however, this practice contributes to the accumulation of drug residues in the food chain, posing potential risks to human health. Restricting antibiotic use in animal feed mitigates the emergence of antibiotic-resistant bacteria, increasing food safety and public health. Owing to their diverse applications in improving animal health, promoting growth, and modulating immunity, plant-derived extracts represent promising alternatives to antibiotics [4–6]. Tannic acid, a widely recognized polyphenol, exhibits antimicrobial properties against *Escherichia coli* and *Helicobacter pylori* through mechanisms such as disrupting microbial metabolism, depriving bacteria of metal ions, and forming complexes with bacterial cell membranes, leading to altered cell wall morphology and increased membrane permeability [7, 8]. Research has demonstrated that tannins reduce parasitic activity in ruminants [9–11] and lower nematode and coccidia excretion in poultry [12]. The antimicrobial and antiparasitic properties of tannins depend primarily on their capacity to bind proteins [7, 13].

Usnic acid, a secondary metabolite produced by lichens with a dibenzofuran structure, has a diverse range of biological activities. Prior to penicillin, usnic acid was a significant lead compound for broad-spectrum antibiotics, primarily because of its activity against Gram-positive bacteria [14]. Its antibacterial properties are attributed to mechanisms such as the inhibition of bacterial cell wall synthesis [15], disruption of bacterial cell membranes [16], and interference with bacterial adenosine triphosphate (ATP) synthesis [17]. Additionally, studies have demonstrated that usnic acid targets protozoan flagellates, inducing cleavage and disruption [18]. These findings highlight the antimicrobial and antiparasitic potential of usnic acid.

Both usnic acid and tannic acid exert their antimicrobial and antiparasitic effects using distinct mechanisms, yet their combined ability to inhibit coccidia, sporozoites, and *C. perfringens* in vitro and to alleviate NE in broilers remains unexplored. This study hypothesizes that usnic acid and tannic acid can inhibit *Eimeria* coccidia and *C. perfringens* while enhancing growth performance and intestinal barrier integrity in NE-infected broilers. This study aimed to assess the inhibitory effects of the combination of usnic acid and tannic acid on *Eimeria* coccidia, sporozoites, and *C. perfringens* through in vitro testing and to evaluate their effects on growth performance, the intestinal immune response, epithelial integrity, and the gut microbiota in vivo following NE infection. To date, this is the first comprehensive study investigating the effects of usnic acid and tannic acid on NE both in vitro and in vivo.

## Materials and methods

### Usnic acid and tannic acid

Usnic acid (extracted from lichens) was obtained from Beijing Centre Biology Co., Ltd. (Beijing, China). Tannic acid (extracted from Chinese gallnut) was obtained from Hubei Chicheng Technology Development Co., Ltd. (Yichang, China).

### Exp. 1: In vitro study

#### Determination of anticoccidial activity

An in vitro anticoccidial activity assay was performed using sporulated strains of *Eimeria tenella* Strain PTMZ, *Eimeria necatrix* Strain PNHZ, *Eimeria maxima * Strain PMHY, and *Eimeria acervulina* Strain PAHY. The assay included 5 treatment groups: (1) the negative control group (NC), (2) the 2 μmol/L diclazuril group (DZ; 98%, Shanghai Macklin Biochemical Science and Technology Co., Ltd., China), (3) the 30 μmol/L usnic acid group (UA), (4) the 90 μmol/L tannic acid group (TA), and (5) the combined 15 μmol/L usnic acid + 45 μmol/L tannic acid group (UTA). Each treatment group was exposed to 2.5 × 10^5^ sporulated coccidian oocysts. After 8 h of treatment, the oocysts were assessed for apoptosis using the ANNEXIN V-FITC/PI Apoptosis Detection Kit (CA1020, Beijing Solarbio Science & Technology Co., Ltd., China) and for mitochondrial membrane potential using the Mitochondrial Membrane Potential Detection Kit (M8650, Beijing Solarbio Science & Technology Co., Ltd., China). A fluidity test kit (ab189819, Abcam Shanghai Trading Co., Ltd., China) was used to detect membrane fluidity. All procedures were performed following the manufacturers’ protocols. Visualization of apoptosis in treated oocysts was conducted using a laser confocal microscope (A1HD25, Nikon, Japan). The experiment was repeated three times to ensure the reliability of the results, and each treatment group consisted of six samples (*n* = 6).

#### Determination of sporozoite activity

Sporozoites were isolated from oocysts using a modified glass bead milling technique as previously described [19]. Each treatment group contained 10^5^ sporozoites, with treatments applied as outlined above. Following 2 h of exposure, sporozoites were assessed for apoptosis using the ANNEXIN V-FITC/PI Apoptosis Detection Kit (CA1020, Beijing Solarbio Science & Technology Co., Ltd., China) and for mitochondrial membrane potential using the Mitochondrial Membrane Potential Detection Kit (M8650, Beijing Solarbio Science & Technology Co., Ltd., China). A fluidity test kit (ab189819, Abcam Shanghai Trading Co., Ltd., China) was used to detect membrane fluidity. All procedures were performed according to the manufacturers’ protocols. The experiment was repeated three times to ensure the reliability of the results, and each treatment group consisted of six samples (*n* = 6).

#### Anti-*C. perfringens* activity

*C. perfringens* (type A, CVCC52) was purchased from the China Veterinary Culture Collection Center. *C. perfringens* cultured to the logarithmic phase at 37 °C under anaerobic conditions. The optical density was adjusted to OD_600_ = 1.0 using a UV spectrophotometer, followed by a tenfold dilution. The diluted culture was spread onto reinforced *Clostridium* agar plates (HB0286, Qingdao High-tech Industrial Park Hopebio Biotechnology Co., Ltd., China). The experiment included five treatment groups: (1) the 100 IU/mL penicillin group (PEN), (2) the 3 mmol/L usnic acid group (UA1), (3) the 88 mmol/L tannic acid group (TA1), (4) the 1.5 mmol/L UA + 44 mmol/L TA group (UTA1), and (5) the acetone solvent control group. The inhibition assay was conducted using the paper disk diffusion method. Each drug-sensitive disk was loaded with 30 µL of the treatment mixture, air-dried completely, and placed on reinforced *Clostridium* agar plates. The plates were incubated for 48 h, after which the diameters of the inhibition zones were measured. The experiment was repeated three times to ensure the reliability of the results, and each treatment group consisted of nine samples (*n* = 9).

### Exp. 2: In vivo study

#### Experimental design

The experiment was conducted at the Poultry Experimental Base of China Agricultural University (Zhuozhou, Hebei, China). A total of 320 hatched (0-d-old) male Shengze 901 chicks with similar body weight (43.82 ± 0.131 g, mean ± standard error) were selected and housed in two-layer cages (1.0 m × 0.7 m × 0.38 m, length × width × height) within a closed chicken house. The chicks were randomly assigned to four treatment groups on the basis of their similar body weights, with five replicates per treatment and 16 chicks per replicate. The treatment groups included (1) the PC group (NE treatment), (2) the SA group (500 mg/kg salinomycin premix + NE treatment), (3) the UA group (300 mg/kg usnic acid + NE treatment), and (4) the UTA group (300 mg/kg usnic acid + 500 mg/kg tannic acid + NE treatment). The experimental period lasted 42 d and was divided into three feeding stages: pre-growth (0–14 d), mid-growth (15–28 d), and late growth (29–42 d). Broilers were provided with feed and water ad libitum, and immunization and management practices adhered to the commercial broiler feeding program. Specifically, the room temperature was maintained at 33–34 °C for the first 3 d and then decreased by 1 °C/d until reaching 20–22 °C, which was maintained until the end of the experiment. Artificial lighting was used in the poultry house. For the first 2 d, continuous 24 h of light was provided, followed by a gradual increase of 1 h of darkness per day until a 4-h dark period was reached. On d 31, the dark period was gradually reduced by 1 h/d until 24 h of continuous light was resumed. The experimental diets were formulated based on the Chinese chicken feeding standard (NY/T 33–2004) [20] and were provided in pellet form. The detailed diet composition is presented in Table 1.

**Table 1:** Ingredients and nutrient composition of the chicken feed used during the trial

Item	**Starter (d 0–14)**	**Grower (d 15–28)**	**Finisher (d 29–42)**
Ingredient, %			
Corn	54.12	58.00	61.79
Soybean meal	32.45	28.00	25.68
Corn gluten meal	5.00	4.00	2.50
Soybean oil	3.00	4.62	4.50
Wheat flour	0.90	0.90	1.05
Calcium hydrogen phosphate	2.00	1.95	1.85
Stone powder	1.00	1.00	1.07
Sodium chloride	0.30	0.30	0.30
L-Lysine hydrochloride (78%)	0.30	0.30	0.36
DL-Methionine (98%)	0.25	0.25	0.19
Threonine	0.10	0.10	0.10
Arginine	0.04	0.04	0.06
Choline chloride (50%)	0.20	0.20	0.20
Mineral premix^1^	0.20	0.20	0.20
Vitamin premix^2^	0.03	0.03	0.03
Phytase 10000	0.01	0.01	0.02
Zeolite	0.10	0.10	0.10
Total	100.00	100.00	100.00
Nutrient content^3^			
Metabolic energy, MJ/kg	12.51	12.97	12.97
Crude protein, %	22.39	20.12	18.5
Lysine, %	1.29	1.17	1.14
Methionine, %	0.61	0.57	0.48
Cystine, %	0.93	0.86	0.75
Threonine, %	0.92	0.83	0.77
Calcium, %	1.08	1.05	1.03
Available phosphorus, %	0.44	0.42	0.40

^1^Composition per kg of mineral premix: copper, 8 g; iron, 40 g; zinc, 55 g; manganese, 60 g; iodine, 750 mg; selenium, 150 mg; and cobalt, 250 mg

^2^Composition per kg of vitamin premix: vitamin A, 50 million IU; vitamin D_3_, 12 million IU; vitamin E, 100,000 IU; vitamin K_3_, 10 g; vitamin B_1_, 8 g; vitamin B_2_, 32 g; vitamin B_6_, 12 g; vitamin B_12_, 100 mg; niacin, 150 g; D-pantothenic acid, 46 g; folic acid, 5 g; biotin, 500 mg

^3^Values calculated on the basis of the experimental diet analysis

#### Construction of an NE model

An NE model was developed in broilers with slight modifications to previously established protocols [21]. At 13 d of age, each group received an oral gavage of 1 mL of a 25-fold attenuated *Eimeria* vaccine suspension (Foshan Zhengdian Biotechnology Co., Ltd., China). From d 17 to 21, the birds were orally administered 1 mL of *C. perfringens* type A CVCC52 (chicken origin) daily at a concentration of 2 × 10^8^ CFU/mL.

#### Sample collection

Each cage was considered an experimental unit. At 28 d of age, one bird with body weight close to the replicate mean was selected for sample collection. Blood was first collected from the wing vein, followed by intramuscular administration of 0.8 mL of Sumianxin into the leg muscle. The birds were euthanized by exsanguination via the jugular vein after anesthesia.

A 1-cm segment of the middle ileum was excised and fixed in 4% paraformaldehyde for histological evaluation. Approximately 2 g of chyme from the middle ileum (midpoint between Meckel’s diverticulum and the ileocecal junction) and cecum was aseptically collected into sterile tubes. Additional tissue and mucosal samples from the middle ileum were snap-frozen in liquid nitrogen and stored at −80 °C for further analyses. Tissue from the distal 4 cm of the ileum was collected for flow cytometric analysis of intestinal immune cells.

#### Growth performance

The body weights of broilers were recorded for each replicate after 12 h of fasting at 13, 21, 28, and 42 d of age. The feed consumption was measured, and the feed conversion ratio (FCR) was calculated.

#### Scoring of intestinal lesions

At 21, 28, and 42 d of age, one bird from each replicate was anesthetized and euthanized via the neck vein via exsanguination. The abdominal cavity was opened, and the duodenum, jejunum, and ileum were isolated. After opening the intestinal lumen and removing the contents, the intestinal segments were examined for lesions. Intestinal lesions were scored independently in a blinded manner based on the criteria described by Dahiya et al. [22].

#### Fecal coccidia count

All broiler feces were collected daily from d 19 to 21 and on d 42 per cage. Fecal coccidia counts were performed using the method described by Long and Rowell [23, 24]. Briefly, after thoroughly mixing the feces from each replicate separately, 2 g of feces from each replicate was placed in a beaker, followed by the addition of 60 mL of saturated saline solution. The mixture was vortexed thoroughly and then filtered through an 80-mesh sieve. The filtrate was then loaded into two counting chambers of a McMaster counting slide, left to settle for 2 min, and subsequently counted. The number of oocysts per gram of feces (oocysts per gram, OPG) was calculated using the following formula:

OPG = Number of oocysts in the counting chamber × 200. The OPG values were log-transformed before statistical analysis.

#### Intestinal morphological indices

Mid-ileum samples fixed in 4% paraformaldehyde were embedded in paraffin, sectioned to a thickness of 5 µm, and stained with hematoxylin and eosin and periodic acid-Schiff reagents. Ileal villus height and crypt depth were measured as outlined by Frankel et al. [25]. In each section, nine intact and straight villi were selected for measurement. The mean villus height, crypt depth, and the villus height to crypt depth ratio (VH:CD) were calculated. Goblet cell counts were also performed, and the number of goblet cells per 100 µm of villus length was recorded.

#### Intestinal barrier and intestinal permeability

Serum diamine oxidase (DAO) and D-lactic acid (D-LA) levels were measured using the Diamine Oxidase Activity Assay Kit (BC1285, Beijing Solarbio Science & Technology Co., Ltd., China) and the D-LA Colorimetric Assay Kit (E-BC-LK002-M, Wuhan Elabscience Biotechnology Co., Ltd., China), respectively. For intestinal mucosa analysis, 0.1 g of mucosal tissue was homogenized in precooled saline at a 1:9 ratio. The protein concentration in the homogenate was determined using a protein quantification kit (PC0020; Beijing Solarbio Science & Technology Co., Ltd., China). Vascular endothelial growth factor (VEGF) and platelet-derived growth factor (PDGF) levels in the mucosal homogenate were quantified using the Chicken VEGF Assay Kit and the Chicken PDGF Assay Kit (Shanghai Enzyme-Linked Biotechnology Co., Ltd., China).

#### Intestinal immunity

Inflammatory cytokine levels in mucosal homogenates were quantified using specific ELISA kits: chicken interleukin 6 (IL-6, SEKCN-0161, Beijing Solarbio Science & Technology Co., Ltd., China), chicken tumor necrosis factor α (TNF-α, SEKCN-0006, Solarbio), interleukin 10 (IL-10, SEKCN-0097, Solarbio), and chicken transforming growth factor β (TGF-β, SEKCN-0005, Solarbio). Secretory immunoglobulin A (sIgA) was also measured using an ELISA kit (YM-A3724, Shanghai Yuanmu Biotechnology Co., Ltd., China). The procedures followed the manufacturers’ protocols, and the results were normalized to the total protein content.

A 4-cm segment from the distal ileum was collected to isolate lymphocytes from the intestinal lamina propria using the method described by Li et al. [26]. The isolated cells were resuspended in 3 mL of RPMI-1640 medium supplemented with 10% fetal bovine serum and adjusted to a concentration of 1 × 10^7^ cells/mL. A 100-µL aliquot of this suspension was stained with monoclonal antibodies specific for chicken CD45, CD3, CD4, CD8, Bu-1, and Mon (Southern Biotechnology Associates Inc., Birmingham, AL, USA) following the manufacturers’ instructions. The stained cells were analyzed using a BD FACSVerse flow cytometer, and the data were processed using FlowJo software.

#### Microbial analysis of the intestinal contents

The mid-ileum and cecal contents from 28-d-old broilers were analyzed using 16S rRNA gene sequencing following the methodology described by Zhang et al. [27]. Bacterial DNA was extracted with the QIAamp Fast DNA Stool Mini Kit (Qiagen, Germany), and the DNA quality and concentration were verified. The V3-V4 hypervariable regions of the 16S rRNA gene were amplified using the universal primers 338 F (5'-ACTCCTACGGGGAGGCAGCA-3') and 806R (5'-GGACTACHVGGGTWTCTAAT-3'). The PCR products were purified, quantified, and pooled for library construction using the TruSeq^®^ DNA PCR-Free Sample Preparation Kit. Libraries were quantified using Qubit and qPCR before sequencing on the HiSeq 2500 PE250 platform. Bioinformatics analysis was performed using QIIME 2 software (version Qiime2-2019.7) and the Majorbio Cloud Platform (www.majorbio.com). Species abundance was mapped at the order and genus levels, and α diversity and β diversity indices were calculated. Significant biomarkers between groups were identified through *t*-test.

#### Short-chain fatty acid (SCFA) determination

A gas chromatograph (GC-2014, Shimadzu Corporation, Kyoto, Japan) equipped with a capillary column (30.0 m × 320 μm × 0.5 μm, Agilent Technologies, Santa Clara, CA, USA) was used to analyze the SCFA contents in the cecal chyme. Briefly, 0.4 g of cecal chyme was placed into a 2-mL sterile centrifuge tube, and 0.5 mL of deionized water was added. The mixture was vortexed, thoroughly shaken, and centrifuged at 15,000 r/min for 10 min at 4 °C. A 0.2-mL aliquot of the supernatant was transferred to a 1.5-mL sterile centrifuge tube, and 50 μL of 25% (w/v) metaphosphoric acid solution containing 2-ethyl butyrate was added. The solution was mixed and allowed to stand at 4 °C overnight to precipitate proteins. The sample was centrifuged again at 15,000 r/min for 10 min at 4 °C, and the supernatant was used for analysis.

### Statistical analysis

Statistical analysis was conducted using one-way ANOVA with Duncan's multiple range test in SPSS 26.0 software. The mortality was assayed using the Chi-square test. Additionally, for data showing significant differences at the initial stage, analysis of covariance (ANCOVA) was applied to account for the initial variation. The results are expressed as the mean ± standard error (SE). For microbiota analysis, correlations between environmental factors and microbial composition were assessed using a Mantel test with the mantel_test() function of the R vegan package. The results from the correlation and Mantel tests were visualized graphically. The Mantel correlation coefficient (*r*) was interpreted as follows: *r* < 0.2—negligible, 0.2 ≤ *r* < 0.4—moderate, and *r* ≥ 0.4—strong. Spearman’s rank correlation coefficient (*r*) was used to assess the correlation between variables. The interpretation of the correlation strength was defined as follows: 0.00 ≤ *r* ≤ 0.10—negligible, 0.10 < *r* ≤ 0.30—weak, 0.30 < *r* ≤ 0.50—moderate, 0.50 < *r* ≤ 0.70—strong, and 0.70 < *r* ≤ 1.00—very strong. Statistical significance thresholds were defined as follows: 0.05 ≤ *P* ≤ 0.1 indicated a trend of difference, *P* < 0.05 indicated statistical significance, and *P* < 0.01 indicated high statistical significance.

## Results

### Inhibitory effects of usnic acid and tannic acid on coccidia, sporozoite and *C. perfringens*

**Fig. 1:**
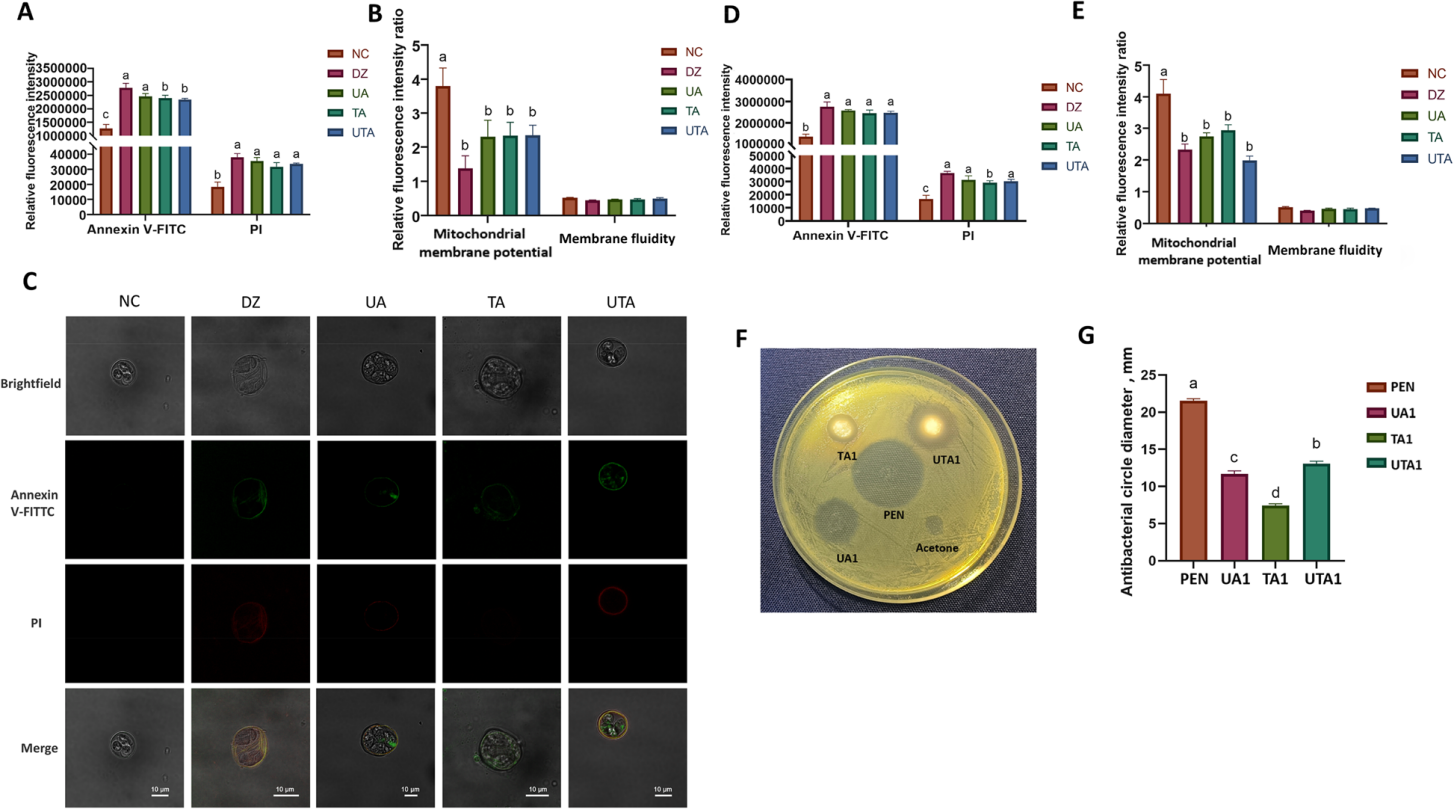
In vitro inhibitory effects of usnic acid and tannic acid on coccidia, sporozoites, and *Clostridium perfringens*. **A** and **D** Impact of usnic acid and tannic acid on the apoptosis of coccidia and sporozoites, respectively, *n* = 6; **B** and **E** Effects of usnic acid and tannic acid on the mitochondrial membrane potential of coccidia and sporozoites, respectively, *n* = 6; **C** Morphology and fluorescence of coccidian oocysts observed under a laser confocal microscope; **F** The inhibitory effects of usnic acid and tannic acid on *C. perfringens* using the paper slide method; **G** The inhibition zone statistics, *n* = 9. NC: control group; DZ: 2 μmol/L diclazuril group; UA: 30 μmol/L usnic acid group; TA: 90 μmol/L tannic acid group; UTA: 15 μmol/L usnic acid + 45 μmol/L tannic acid group; PEN: 100 IU/mL penicillin group; UA1: the 3 mmol/L UA group; TA1: the 88 mmol/L TA group; UTA1: the 1.5 mmol/L UA + 44 mmol/L TA group. ^a–d^Different letters indicate significant differences (*P* < 0.05)

Figure 1A illustrates the apoptotic status of coccidian oocysts, where green fluorescence represents early apoptosis, and late apoptotic cells exhibit both red and green fluorescence. The Annexin V-FITC and PI fluorescence intensities were significantly greater in the DZ, UA, TA, and UTA groups than in the NC group (*P* < 0.05). Figure 1B shows a reduction in the mitochondrial membrane potential of coccidian oocysts in the DZ, UA, TA, and UTA groups compared with those in the NC group (*P* < 0.05). There was no significant effect on cell membrane fluidity. Figure 1C shows that the oocysts in the NC group maintained intact structures with clear cell walls, whereas the oocysts in the DZ, UA, TA, and UTA groups displayed structural damage, including crumpled edges and disorganized internal structures, which was consistent with the observed apoptosis results. Sporozoites followed a similar trend to that of the oocysts. Figure 1D shows increased Annexin V-FITC and PI fluorescence intensities in sporozoites from the DZ, UA, TA, and UTA groups compared with those from the NC group (*P* < 0.05). Similarly, Fig. 1E shows a lower mitochondrial membrane potential in sporozoites in DZ, UA, TA and UTA groups than in those in the NC group (*P* < 0.05). Figures 1F and G present the results of the *C. perfringens* inhibition test, revealing that the UA1, TA1, and UTA1 groups exhibited inhibitory effects against *C. perfringens*. The size of the inhibition zone decreased in the following order: UTA1 group > UA1 group > TA1 group.

### Effects of usnic acid and tannic acid on the performance of broilers with NE

The effects of UA and TA on the production performance of broilers with NE are summarized in Table 2. Compared with the PC group, the SA group presented significantly greater body weight at 13 d and 21 d, greater body weight gain from 14 to 21 d, greater feed consumption during 0–21 d and 14–21 d, and a lower FCR at 0–21 d, 14–21 d, and 0–28 d (*P* < 0.05). Similarly, the UA group presented significantly increased body weight at 13 d and 21 d and increased feed consumption during 0–21 d and 14–21 d (*P* < 0.05). Compared with the PC group, the UTA group presented significantly improved body weight at 13 d and 21 d, increased body weight gain from 14 to 21 d, increased feed consumption from 0 to 21 d, and reduced FCR during 0–13 d and 0–21 d (*P* < 0.05). Mortality and FCR at 22–28 d, 29–42 d, and 0–42 d did not differ significantly among the treatment groups.

**Table 2:** Effects of usnic acid and tannic acid on broiler growth performance with NE

Items	PC	SA	UA	UTA	*P*-value
Body weight, g					
0 d	43.78 ± 0.281	43.84 ± 0.327	43.95 ± 0.157	43.70 ± 0.327	0.934
13 d	461.20 ± 2.524^b^	493.75 ± 10.097^a^	482.13 ± 2.822^a^	479.56 ± 4.298^a^	0.009
21 d	904.44 ± 9.626^c^	1,013.37 ± 10.065^a^	950.18 ± 13.264^b^	971.49 ± 8.972^b^	< 0.01
28 d	1,587.56 ± 15.922	1,684.08 ± 13.269	1,636.01 ± 27.208	1,637.11 ± 28.413	0.054
42 d	3,059.37 ± 40.471	3,185.31 ± 59.767	3,116.81 ± 42.648	3,177.57 ± 58.160	0.376
Body weight gain, g					
14–21 d	443.24 ± 10.346^ cd^	533.34 ± 7.912^a^	468.05 ± 14.550^bc^	491.92 ± 10.835^b^	0.005
22–28 d	672.86 ± 12.672	670.71 ± 7.195	685.83 ± 19.840	665.62 ± 21.679	0.446
29–42 d	1,471.81 ± 30.384	1501.24 ± 53.323	1,485.70 ± 22.826	1,480.95 ± 56.149	0.971
Feed consumption, g					
0–13 d	517.51 ± 2.837	532.94 ± 7.856	534.03 ± 2.627	526.34 ± 5.477	0.132
14–21 d	668.14 ± 7.965^b^	736.84 ± 13.863^a^	722.92 ± 17.524^a^	710.29 ± 17.810^ab^	0.027
0–21 d	1,185.66 ± 8.935^b^	1,271.14 ± 19.371^a^	1,256.94 ± 18.041^a^	1,236.63 ± 18.408^a^	0.012
22–28 d	985.12 ± 17.387	960.67 ± 5.164	996.14 ± 24.730	979.00 ± 26.786	0.186
0–28 d	2,170.78 ± 23.451	2,228.57 ± 21.359	2,253.08 ± 42.011	2,215.63 ± 35.970	0.351
29–42 d	2,479.74 ± 50.071	2,514.67 ± 66.336	2,473.12 ± 12.986	2,431.31 ± 29.590	0.700
0–42 d	4,650.52 ± 70.459	4,741.87 ± 74.600	4,694.68 ± 40.249	4,730.05 ± 81.642	0.776
FCR					
0–13 d	1.240 ± 0.0022^a^	1.222 ± 0.0076^ab^	1.219 ± 0.0067^ab^	1.208 ± 0.0086^b^	0.028
14–21 d	1.509 ± 0.0203^a^	1.382 ± 0.0169^c^	1.498 ± 0.0206^ab^	1.474 ± 0.0195^ab^	0.001
0–21 d	1.378 ± 0.0078^a^	1.311 ± 0.0082^c^	1.365 ± 0.0055^ab^	1.333 ± 0.0020^bc^	0.004
22–28 d	1.456 ± 0.0140	1.428 ± 0.0132	1.466 ± 0.0029	1.463 ± 0.0060	0.129
0–28 d	1.406 ± 0.0068^a^	1.359 ± 0.0057^b^	1.400 ± 0.0061^a^	1.391 ± 0.0114^a^	0.003
29–42 d	1.685 ± 0.0050	1.654 ± 0.0089	1.661 ± 0.0222	1.673 ± 0.0352	0.816
0–42 d	1.542 ± 0.0044	1.510 ± 0.0107	1.529 ± 0.0161	1.510 ± 0.0125	0.145
Mortality during challenge,%	3.75 ± 1.531	2.50 ± 1.531	5.00 ± 2.339	2.50 ± 2.500	0.456

Data are presented as the mean ± standard error. ^a^^–d^ Different letters indicate significant differences (*P* < 0.05). PC: positive control group; SA: 500 mg/kg salinomycin group; UA: 300 mg/kg usnic acid group; UTA: 500 mg/kg tannic acid + 300 mg/kg usnic acid group. *n* = 5

### Effects of usnic acid and tannic acid on the intestinal lesion scores of broilers with NE

The effects on intestinal lesion scores are presented in Table 3. The SA group presented significantly lower lesion scores in the jejunum and ileum at 28 d (*P* < 0.05). The UA and UTA groups had significantly lower ileum lesion scores at 28 d (*P* < 0.05). Additionally, the UTA group had significantly lower jejunum lesion scores at 28 d than the PC group (*P* < 0.05).

**Table 3:** Effects of usnic acid and tannic acid on intestinal lesion scores in broilers with NE

Items	PC	SA	UA	UTA	*P*-value
21 d					
Duodenum	0.84 ± 0.181	0.71 ± 0.246	0.33 ± 0.119	0.36 ± 0.019	0.106
Jejunum	0.97 ± 0.033	0.79 ± 0.257	0.48 ± 0.156	0.59 ± 0.127	0.201
Ileum	0.85 ± 0.092	0.71 ± 0.099	0.63 ± 0.175	0.51 ± 0.131	0.326
28 d					
Duodenum	0.83 ± 0.370	0.05 ± 0.023	0.36 ± 0.091	0.35 ± 0.102	0.086
Jejunum	1.62 ± 0.255^a^	0.80 ± 0.243^b^	1.02 ± 0.242^ab^	0.52 ± 0.161^b^	0.023
Ileum	1.29 ± 0.245^a^	0.53 ± 0.154^b^	0.47 ± 0.031^b^	0.39 ± 0.113^b^	0.003
42 d					
Duodenum	0.52 ± 0.082	0.48 ± 0.109	0.52 ± 0.116	0.54 ± 0.130	0.981
Jejunum	1.48 ± 0.275	0.89 ± 0.159	0.71 ± 0.169	0.82 ± 0.132	0.051
Ileum	1.15 ± 0.295	0.68 ± 0.145	0.60 ± 0.063	0.48 ± 0.073	0.064

Data are presented as the mean ± standard error. ^a,b^Different letters indicate significant differences. PC: positive control group; SA: 500 mg/kg salinomycin group; UA: 300 mg/kg usnic acid group; UTA: 500 mg/kg tannic acid + 300 mg/kg usnic acid group. *n* = 5

### Effects of usnic acid and tannic acid on the number of coccidian oocysts in the feces of broilers with NE

**Fig. 2:**
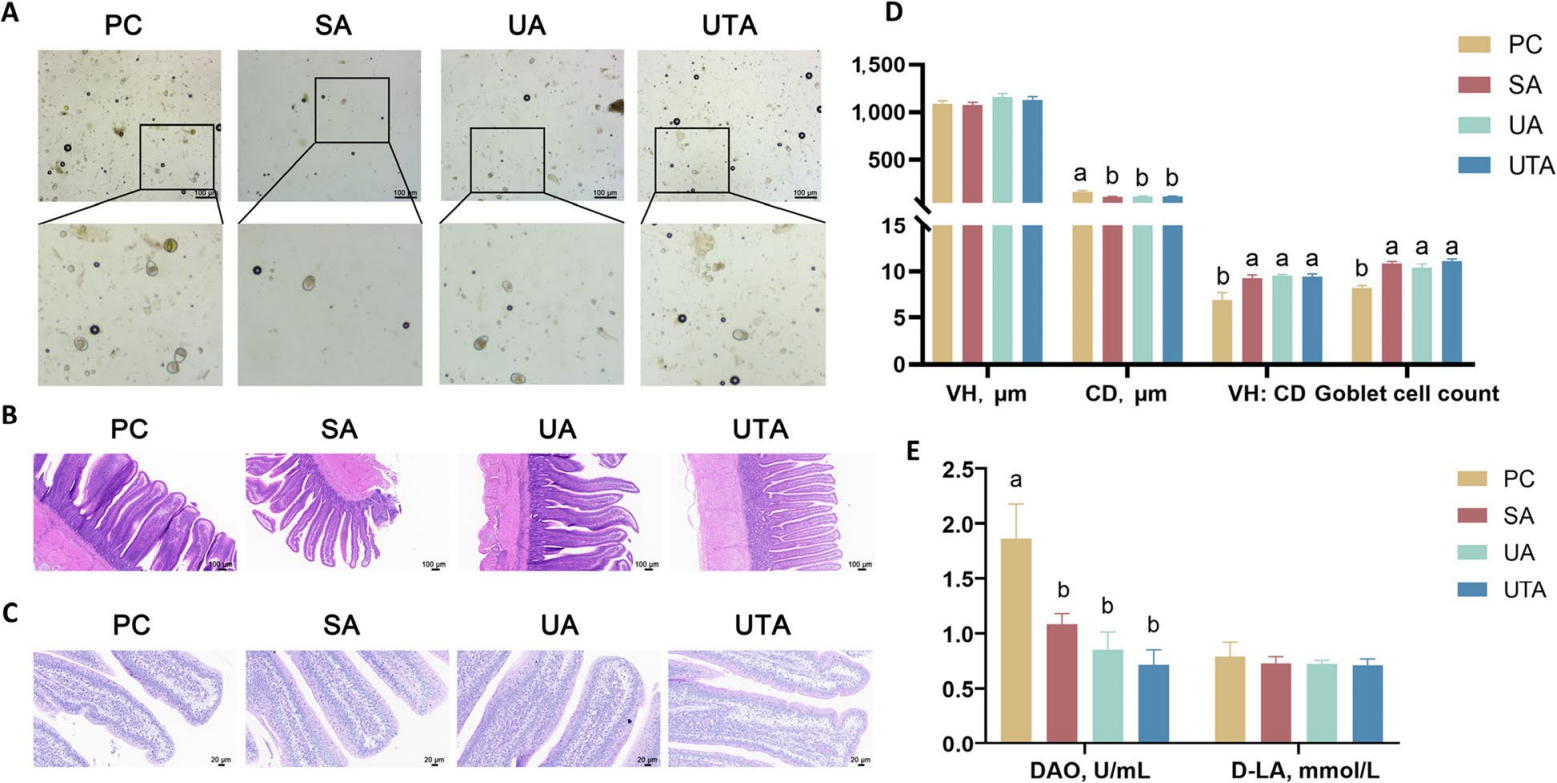
Effects of usnic acid and tannic acid on coccidian oocysts in feces, intestinal morphology, and barrier function in broilers with necrotic enteritis (NE). **A** Coccidian oocysts in feces under a light microscope; **B** and **C** Intestinal villus morphology and goblet cell images; **D** Intestinal villus morphology and goblet cell count; **E** Serum DAO and D-LA content. ^a,b^Different letters indicate significant differences (*P* < 0.05). PC: positive control group; SA: 500 mg/kg salinomycin group; UA: 300 mg/kg usnic acid group; UTA: 500 mg/kg tannic acid + 300 mg/kg usnic acid group. *n* = 5

Table 4 presents the counts of coccidian oocysts in the feces. Figure 2A shows the fecal coccidian oocysts observed under a light microscope. Compared with the PC group, the SA and UTA groups presented significantly lower fecal coccidian oocyst counts from 19 to 21 d and at 42 d (*P* < 0.05). Similarly, compared with the PC group, the UA group presented significantly lower coccidian oocyst counts in feces from 19 to 21 d (*P* < 0.05).

**Table 4:** Effects of usnic acid and tannic acid on the number of coccidia oocysts in the feces of broilers with NE

Items	PC	SA	UA	UTA	*P*-value
19 d	4.81 ± 0.016^a^	4.04 ± 0.105^c^	4.62 ± 0.031^b^	4.49 ± 0.038^b^	< 0.001
20 d	4.85 ± 0.047^a^	3.83 ± 0.055^c^	4.60 ± 0.027^b^	4.49 ± 0.021^b^	< 0.001
21 d	4.50 ± 0.026^a^	3.26 ± 0.104^c^	4.22 ± 0.032^b^	4.23 ± 0.099^b^	< 0.001
42 d	3.77 ± 0.032^a^	3.12 ± 0.259^b^	3.44 ± 0.119^ab^	3.18 ± 0.142^b^	0.016

Data are presented as the mean ± standard error. ^a^^–^^c^Different letters indicate significant differences. PC: positive control group; SA: 500 mg/kg salinomycin group; UA: 300 mg/kg usnic acid group; UTA: 500 mg/kg tannic acid + 300 mg/kg usnic acid group. The data presented in Table 4 have undergone log-transformation. *n* = 5

### Effects of usnic acid and tannic acid on intestinal villus morphology and intestinal barrier function in broilers with NE

Figures 2B and C show the intestinal villus morphology and goblet cell images, respectively. Measurements of intestinal morphology and counting of goblet cells revealed no significant difference in villus height across all groups; however, crypt depth was significantly lower in the SA, UA, and UTA groups than in the PC group (*P* < 0.05). Additionally, the VH:CD ratio and goblet cell count were significantly greater in the SA, UA, and UTA groups than in the PC group (Fig. 2D, *P*< 0.05). Figure 2E shows the DAO and D-LA levels in the serum. DAO activity was significantly lower in the SA, UA, and UTA groups than in the PC group (*P* < 0.05), indicating improved intestinal barrier integrity compared with that in the PC group, while no significant differences in the serum D-LA content were detected among the groups.

### Effects of usnic acid and tannic acid on intestinal immune function in broilers with NE

**Fig. 3:**
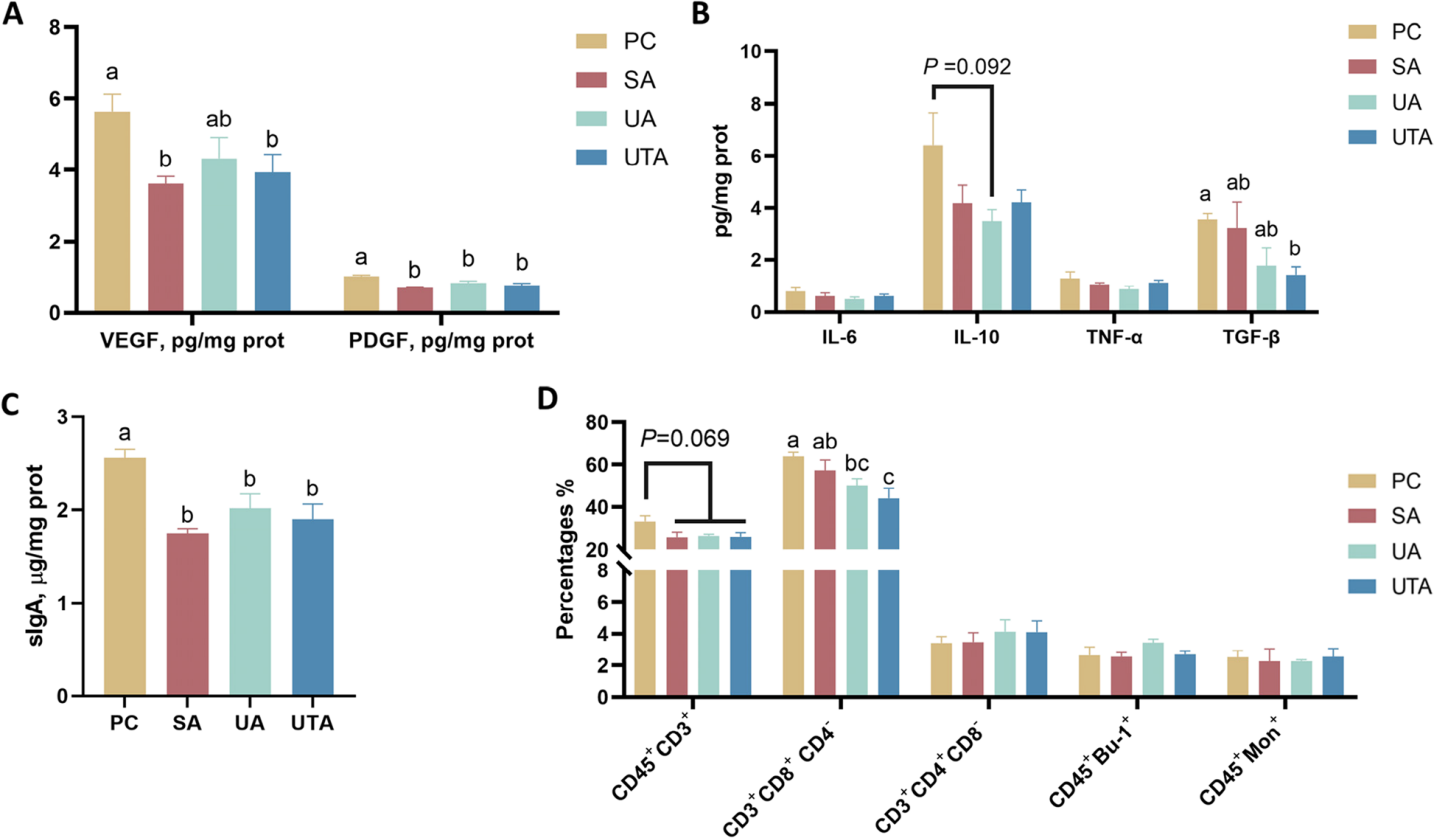
Effects of usnic acid and tannic acid on intestinal immune function in broilers with NE. **A** VEGF and PDGF content in the intestinal mucosa. **B** Inflammatory factor content in the intestinal mucosa; **C** sIgA content in the intestinal mucosa; **D** Immune cell content in the intestines, analyzed by flow cytometry. ^a^^–^^c^Different letters indicate significant differences (*P* < 0.05). PC: positive control group; SA: 500 mg/kg salinomycin group; UA: 300 mg/kg usnic acid group; UTA: 500 mg/kg tannic acid + 300 mg/kg usnic acid group. *n* = 5

The intestinal coagulation function and immunity-related indices were evaluated using enzyme immunoassays and flow cytometry. Figure 3A shows that the VEGF content in the intestinal mucosa was significantly lower in the SA and UTA groups (*P* < 0.05), whereas the PDGF content was significantly lower in the SA, UA, and UTA groups than in the PC group (*P* < 0.05). Figure 3B shows that, compared with the PC group, the UA group tended to have a lower IL-10 content in the intestinal mucosa (*P* = 0.092), whereas the UTA group had significantly lower TGF-β levels (*P* < 0.05). There were no significant differences in the IL-6 or TNF-α levels among the groups. Figure 3C shows that the sIgA content in the mucosa was significantly lower in the SA, UA, and UTA groups than in the PC group (*P* < 0.05). Figure 3D shows a trend toward a reduced proportion of CD45^+^CD3^+^ cells in the SA, UA, and UTA groups compared with the PC group (*P* = 0.069). The proportion of CD3^+^CD8^+^CD4^–^ cells was significantly lower in the UA and UTA groups than in the PC and SA groups (*P* < 0.05). There were no significant differences in the proportions of CD3^+^CD4^+^ CD8^–^, CD45^+^Bu-1^+^, or CD45^+^Mon^+^ cells among the groups.

### Effects of usnic acid and tannic acid on intestinal microorganisms in broilers with NE

**Fig. 4:**
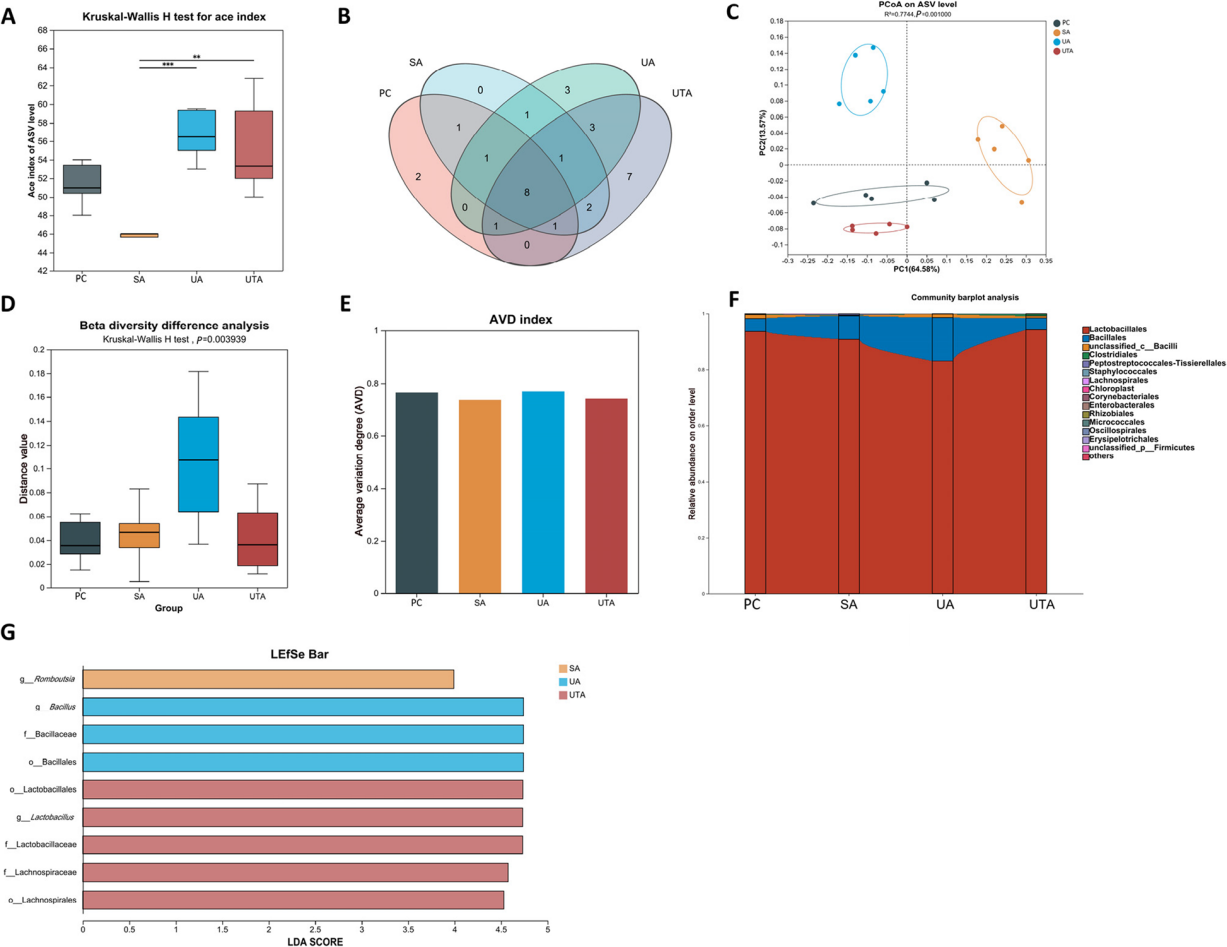
Effects of usnic acid and tannic acid on ileal microorganisms in broilers with NE. **A** α diversity index; **B** Venn plot for species differences at the genus level; **C** PCoA plot; **D** Differences in β diversity; **E** AVD index of species stability; **F** Ileal microbial abundance at the order level; **G** LEfSe analysis of ileal microbes. *P* < 0.01 is marked as **, and *P* < 0.001 is marked as ***. PC: positive control group; SA: 500 mg/kg salinomycin group; UA: 300 mg/kg usnic acid group; UTA: 500 mg/kg tannic acid + 300 mg/kg usnic acid group. *n* = 5

Figure 4 presents the effects of UA and TA on ileal microorganisms. Figure 4A shows that the ACE index, which reflects α diversity, was significantly greater in the UA and UTA groups than in the SA group (*P* < 0.01). Figure 4B presents a Venn diagram at the genus level, showing two unique genera in the PC group, none in the SA group, and three and seven unique genera in the UA and UTA groups, respectively. Figures 4C and D highlight the significant differences in β diversity and sample composition among the groups (*P* < 0.01). Figure 4E shows lower AVD indices in the SA and UTA groups. Figure 4F shows that Lactobacillales represented more than 80% of the ileal microbial community, followed by Bacillales, Bacilli, and Clostridiales. LEfSe analysis results (Fig. 4G, LDA > 3.9) revealed *Romboutsia* as dominant in the SA group; Bacillales, Bacillaceae, and *Bacillus* as dominant in the UA group; and Lactobacillales and Lachnospirales as dominant in the UTA group.

**Fig. 5:**
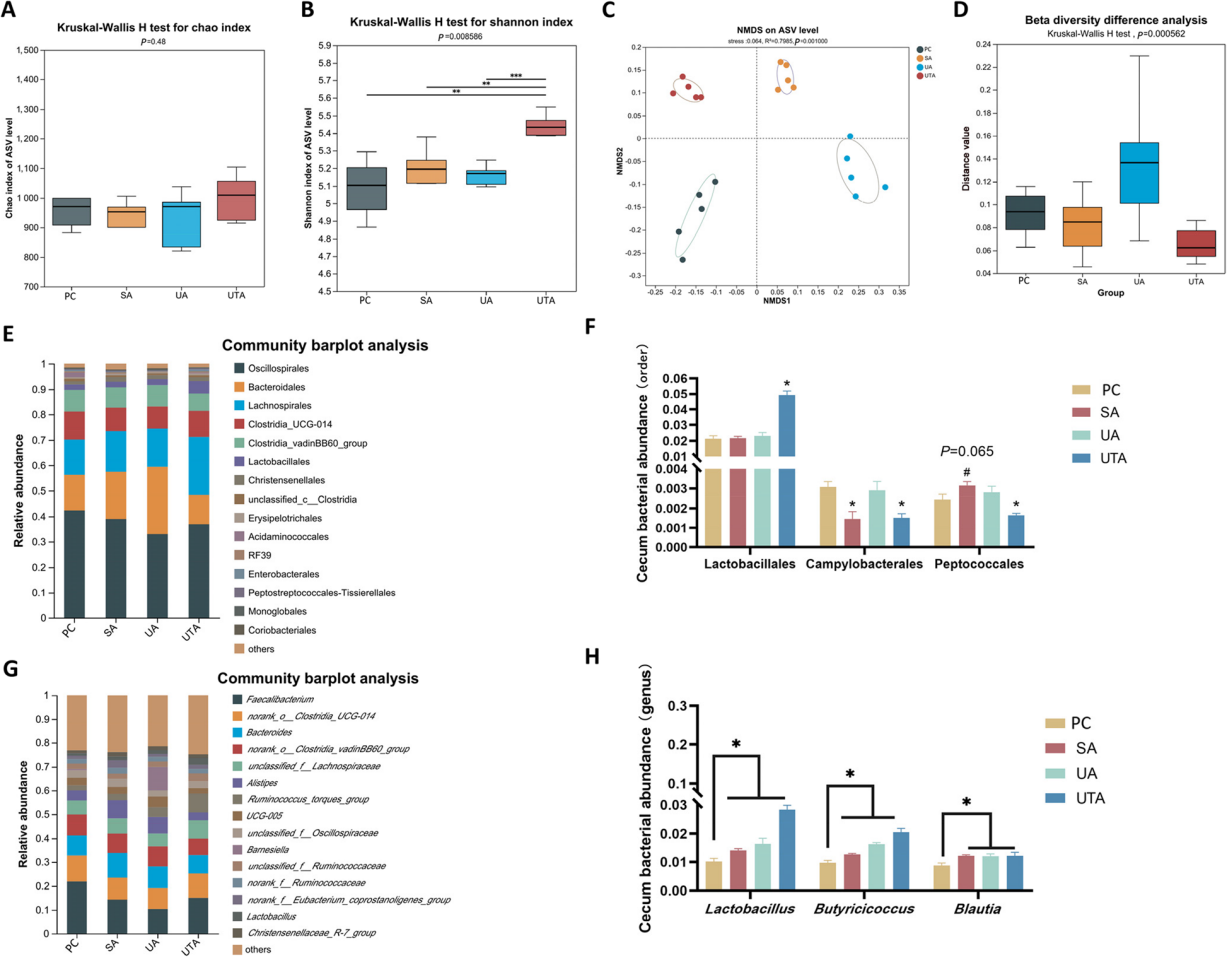
Effects of usnic acid and tannic acid on cecum microorganisms in broilers with NE. **A** and **B** Chao and Shannon indices of α diversity, respectively; **C** NMDS plot of species distribution; **D** Differences in β diversity; **E** shows the abundance of cecum microorganisms at the order level; **F** *t*-test results for species differences at the order level; **G** Abundance of cecum microorganisms at the genus level; **H** *t*-test results for species differences at the genus level. *P* < 0.05 is marked as *, *P* < 0.01 as **, *P* < 0.001 as ***, and 0.05 ≤ *P* ≤ 0.1 as #. PC: positive control group; SA: 500 mg/kg salinomycin group; UA: 300 mg/kg usnic acid group; UTA: 500 mg/kg tannic acid + 300 mg/kg usnic acid group. *n* = 5

Figure 5 presents the results of the cecal microbial analysis. Figures 5A and B show no significant differences in the Chao index (α diversity) among the treatment groups, whereas the Shannon index (α diversity) was significantly greater in the UTA group than in the other groups (*P* < 0.01). Figures 5C and D reveal substantial differences in sample composition, with significant β diversity variations among the treatment groups (*P* < 0.001). Figure 5E shows the relative abundance of species at the order level, with Oscillospirales, Bacteroidales, Lachnospirales, and Clostridia UCG-014 being predominant. Further analysis, as shown in Fig. 5F, revealed that, compared with the PC group, the UTA group presented a significantly greater relative abundance of Lactobacillales and notably lower abundances of Campylobacterales and Peptococcales (*P* < 0.05). Compared with the PC group, the SA group presented a significantly lower abundance of Campylobacterales (*P* < 0.05) and a trend toward a greater abundance of Peptococcales (*P* = 0.065). Figure 5G shows that *Faecalibacterium*, *norank_o_Clostridia_UCG-01*, and *Bacteroides* were the dominant genera. Differential species analysis (Fig. 5H) revealed that the relative abundances of *Lactobacillus*, *Butyricicoccus*, and *Blautia* were significantly greater in the SA, UA, and UTA groups than in the PC group (*P* < 0.05).

### Effects of usnic acid and tannic acid on SCFA content in the cecum of broilers with NE

Table 5 summarizes the analysis of SCFA content in the cecum. Compared with the PC group, the SA group presented significantly increased acetate concentrations, whereas the UTA group presented significantly elevated acetate and butyrate concentrations (*P* < 0.05).

**Table 5:** Effects of usnic acid and tannic acid on short-chain fatty acid content in the cecum of broilers with NE

Item	**PC**	**SA**	**UA**	**UTA**	***P*-value**
Acetate	46.10 ± 3.791^b^	67.99 ± 9.605^a^	56.09 ± 2.756^ab^	72.33 ± 6.046^a^	0.041
Propionate	9.72 ± 1.465	9.39 ± 2.482	12.76 ± 1.226	8.40 ± 0.514	0.344
Isobutyrate	0.89 ± 0.202	0.79 ± 0.048	0.82 ± 0.052	0.77 ± 0.099	0.908
Butyrate	11.59 ± 0.916^b^	14.60 ± 2.045^ab^	18.03 ± 3.100^ab^	20.31 ± 0.879^a^	0.048
Isovalerate	1.30 ± 0.319	1.02 ± 0.079	1.33 ± 0.184	1.14 ± 0.119	0.654
Valerate	0.84 ± 0.094	0.79 ± 0.053	1.04 ± 0.054	0.96 ± 0.062	0.106

Data are presented as the mean ± standard error. ^a,b^Different letters indicate significant differences. PC: positive control group; SA: 500 mg/kg salinomycin group; UA: 300 mg/kg usnic acid group; UTA: 500 mg/kg tannic acid + 300 mg/kg usnic acid group. *n* = 5

### Correlation analysis of indicators

**Fig. 6:**
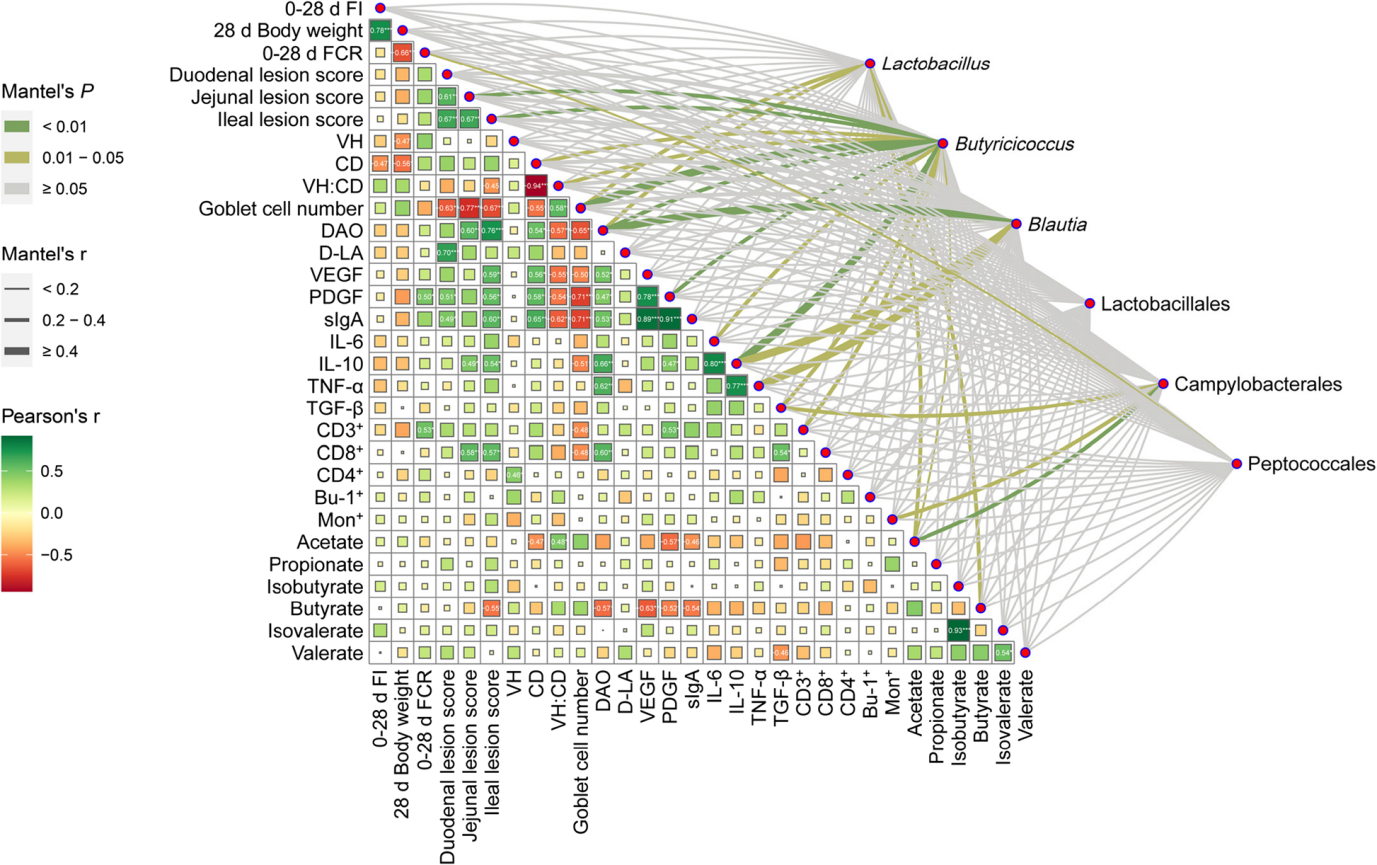
Heat map showing correlations between intestinal microbiota, growth performance, intestinal immunity, and barrier-related parameters, with each cell representing the correlation coefficient and magnitude. Connecting lines between differentially enriched microbial genera in the ileum and cecum correlate with the corresponding environmental factors, with line thickness and color indicating the strength and significance of the correlation. * indicates *P* < 0.05; ** indicates *P* < 0.01; *** indicates *P* < 0.001

Spearman correlation analysis was conducted to evaluate the relationships between broiler growth performance (28 d body weight, 0–28 d feed intake, and 0–28 d FCR), intestinal health indicators (villus morphology, immunity, the intestinal barrier, and coagulation function), and intestinal bacteria with differential abundance (Fig. 6). The body weight at 28 d was significantly negatively correlated with the 0–28 d FCR (*P* < 0.01, *r* = −0.66), negatively correlated with CD (*P* < 0.05, *r* = −0.56), and positively correlated with 0–28 d FI (*P* < 0.001, *r* = 0.78). The 0–28 d FCR was significantly correlated with CD3^+^ (*P* < 0.05, *r* = 0.53). The duodenal lesion score was positively correlated with both jejunal (*P* < 0.01, *r* = 0.61) and ileal lesion scores (*P* < 0.01, *r* = 0.67). Additionally, the duodenal lesion score was strongly significantly negatively correlated with the number of goblet cells (*P* < 0.01, *r* = −0.63), highly significantly positively correlated with D-LA (*P* < 0.001, *r* = 0.70), and significantly positively correlated with PDGF (*P* < 0.05, *r* = 0.51). The jejunal lesion score was highly significantly positively correlated with DAO (*P* < 0.01, *r* = 0.60) and CD8^+^ (*P* < 0.01, *r* = 0.58), and significantly negative correlation with the number of goblet cells (*P* < 0.001, *r* = −0.77). The ileal lesion score showed a highly significant negative correlation with the number of goblet cells (*P* < 0.01, *r* = −0.67) and butyrate(*P* < 0.05, *r* = −0.55), while it also demonstrated a highly significant positive correlation with DAO (*P* < 0.001, *r* = 0.76), VEGF (*P* < 0.01, *r* = 0.59), PDGF (*P* < 0.01, *r* = 0.56), sIgA (*P* < 0.01, *r* = 0.60), and CD8^+^ (*P* < 0.01, *r* = 0.57) and a significant positive correlation with IL-10 (*P* < 0.05, *r* = 0.54). The VH:CD ratio was highly positively correlated with the number of goblet cells (*P* < 0.01, *r* = 0.58), highly negatively correlated with DAO (*P* < 0.01, *r* = −0.57) and sIgA (*P* < 0.01, *r* = −0.62), and significantly negatively correlated with VEGF (*P* < 0.05, *r* = −0.55) and PDGF (*P* < 0.05, *r* = −0.54). VEGF showed a highly significant positive correlation with PDGF (*P* < 0.001, r = 0.78) and sIgA (*P* < 0.001, *r* = 0.89), and a significant negative correlation with butyrate (*P* < 0.05, *r* = −0.63). PDGF was highly significantly positively correlated with sIgA (*P* < 0.001, *r* = 0.91), significantly positively correlated with CD3^+^ (*P* < 0.05, *r* = 0.53), and significantly negatively correlated with butyrate (*P* < 0.05, *r* = −0.52) and acetate (*P* < 0.05, *r* = −0.57). *Butyricicoccus* was highly significantly positively correlated with the number of goblet cells (*P* < 0.01, *r* ≥ 0.4), significantly positively correlated with IL-10 (*P* < 0.05, *r* ≥ 0.4). *Blautia* was significantly positively correlated with DAO (*P* < 0.01, *r* ≥ 0.4), IL-10 (*P* < 0.05, *r* ≥ 0.4), TNF-α (*P* < 0.05, *r* ≥ 0.4).

## Discussions

### Usnic acid and tannic acid inhibited the proliferation of *C. perfringens* and the activity of coccidia and sporozoite

This study demonstrated the inhibitory effects of usnic acid and tannic acid both individually and in combination on two primary pathogens associated with NE: coccidia and *C. perfringens*. The results suggest that usnic acid and tannic acid, as natural plant-derived compounds, induce apoptosis in coccidia and sporozoites by reducing the mitochondrial membrane potential, elucidating a potential mechanism for their inhibitory activity. In vivo experiments further revealed that the combination of usnic acid and tannic acid significantly decreased fecal coccidian oocyst counts compared with usnic acid alone, underscoring the synergistic advantage of combining these compounds. These findings align with previous research by Jang et al. [28], who reported a notable reduction in fecal oocyst counts in chickens fed green tea-based diets following infection with *Eimeria maxima*. Usnic acid exerts its effects by disrupting tocopherol biosynthesis [29], interfering with the syncytium-to-kinetochore transition [30], and compromising pre-flagellar body structures [31]. In contrast, tannic acid directly targets parasitic stages such as free-living larvae and parasitized adults by inhibiting larval development, reducing larval colonization, and impairing egg laying and motility [32]. Additionally, tannic acid enhances host immune function and infection resistance through indirect pathways [33]. Together, these results underscore the potential of usnic acid and tannic acid and highlight that usnic acid and tannic acid employ distinct mechanisms to inhibit parasitic activity.

In bacteriostatic tests, usnic acid and tannic acid had significant inhibitory effects on *C. perfringens*, with usnic acid exhibiting a stronger inhibitory effect than tannic acid. The antimicrobial activity of usnic acid is attributed to its dibenzofuran structure, which interferes with RNA and DNA synthesis [18]. Usnic acid penetrates bacterial cells as an ion, disrupts adenosine triphosphate (ATP) synthesis [17], and increases bacterial sensitivity to environmental factors, leading to membrane damage [16]. In contrast, the antimicrobial mechanisms of tannic acid involve disrupting bacterial metabolism, inhibiting the uptake of nutrients such as amino acids, impairing adhesion, and compromising membrane integrity [33, 34]. In summary, both usnic acid and tannic acid display potent anticoccidial properties and effectively inhibit the growth of *C. perfringens*, thereby enhancing production performance in broilers. Despite the promising outcomes observed with the combined use of usnic acid and tannic acid, further studies are needed to fully elucidate the mechanisms driving their synergistic effects.

### Usnic acid and tannic acid enhance growth performance by improving intestinal villus morphology and barrier integrity and alleviating inflammation

The morphology of the intestinal villi and the integrity of the intestinal barrier are essential factors affecting animal performance. Several studies have indicated that NE negatively impacts poultry growth production by causing damage to the intestinal mucosa and villi, leading to significant economic losses [35, 36]. These results are consistent with those of the present study. The increase in crypt depth observed in broilers affected by NE may indicate a compensatory repair mechanism in the intestine, wherein crypt cell proliferation and repair are stimulated in response to intestinal injury. This observation is in line with previous studies [21, 37]. Supplementation with usnic acid, either alone or combined with tannic acid, enhanced the morphological structure of the intestinal villi, improved the VH:CD ratio, and increased the surface area available for nutrient absorption. These changes likely contributed to the improved growth performance. The intestinal mucus plays a crucial role in safeguarding the epithelial surface against pathogens, promoting commensal bacteria colonization, maintaining a favorable digestive environment, and facilitating nutrient transport [38]. The mucus layer is produced and maintained by goblet cells, which were assessed in this study. NE led to a significant reduction in the number of goblet cells in the intestine, which is consistent with previous findings [39, 40]. A decrease in the number of goblet cells results in reduced mucin secretion, increasing the host’s vulnerability to bacterial infections [41]. Intestinal permeability increases during mucosal disruption and inflammation. In this study, serum DAO activity was elevated in NE broilers, indicating compromised intestinal barrier integrity, which is in line with earlier studies [42]. The combined supplementation of usnic acid and tannic acid reduced serum DAO levels, suggesting a positive impact on intestinal barrier integrity in NE-challenged broilers. While there is no direct evidence in the literature regarding the impact of usnic acid on intestinal barrier function, recent studies have shown that tannic acid supplementation significantly lowers serum DAO levels in heat-stressed broilers [43]. These results highlight the beneficial effects of tannic acid on intestinal barrier function, although further research is needed to understand the mechanisms by which usnic acid and tannic acid improve intestinal barrier integrity. In conclusion, NE-induced damage to intestinal villi and a reduction in the number of goblet cells are closely linked to impaired growth performance. The ability of usnic acid and tannic acid to improve villus morphology, increase goblet cell numbers, and preserve intestinal barrier integrity highlights their potential to alleviate NE-induced intestinal damage and improve poultry growth performance.

VEGF is essential for increasing vascular endothelial cell permeability and promoting angiogenesis [44]. High VEGF expression in vivo typically indicates vascular endothelial damage, which can trigger platelet aggregation, capillary leakage, and localized thrombosis, thus worsening mucosal congestion [45]. PDGF, a peptide regulator released during platelet activation alongside factors such as TGF-β, promotes connective tissue repair, enhances the adhesion of neutrophils, monocytes, and eosinophils, and induces substances with vasodilatory and antiplatelet effects. TGF-β is widely recognized for its role in wound healing; supporting cell differentiation, proliferation, and migration; and facilitating tissue regeneration and repair [46]. In this study, the increased expression of VEGF and PDGF in the intestinal mucosa of NE-challenged broilers suggested increased microvascular permeability and possible local tissue congestion. This likely triggered the expression of TGF-β to repair the damaged intestinal mucosa. Importantly, the combination of usnic acid and tannic acid effectively lowered VEGF, PDGF, and TGF-β levels, indicating that this combined supplementation reduced microvascular permeability and inflammation within the mucosa. This reduction likely limits the infiltration of inflammatory cells, focal hemorrhage, and blood vessel regeneration, thus reducing congestion, edema, ulceration, and hemorrhage in the intestinal mucosa. The combination of usnic acid and tannic acid was more effective in reducing intestinal mucosal congestion than usnic acid alone, likely due to the astringent properties of tannic acid. Tannic acid interacts with tissue proteins, forming a protective layer by precipitating proteins on ulcer surfaces, thus safeguarding the intestinal mucosa from additional damage [47]. However, further pathological investigations are needed to verify these findings and explore the underlying mechanisms in greater detail.

When the intestinal mucus barrier is compromised, pathogens can directly interact with epithelial cells, triggering immune responses. CD3 is a T-cell marker expressed on both T helper and T cytotoxic cells, while CD8^+^ T cells are essential components of the adaptive immune system and play a key role in defending against foreign organisms [48, 49]. In this study, NE increased the proportion of CD3^+^ and CD8^+^ cells, indicating enhanced immune activation in response to intestinal damage. Additionally, NE was found to increase the levels of inflammatory factors and sIgA, contributing to intestinal inflammatory responses, which aligns with the findings of previous studies [41, 50, 51]. However, UTA significantly inhibited inflammation. Previous research has shown that usnic acid can effectively reduce inflammatory factor expression in lipopolysaccharide-stimulated RAW 264.7 macrophages [52, 53], and its anti-inflammatory effects have been applied in conditions such as acute lung injury and acute respiratory distress syndrome [54]. Similarly, tannins are widely recognized for their anti-inflammatory properties [55]. In the present study, in addition to the anti-inflammatory effects of usnic acid and tannic acid, the reduction in the inflammatory response may also be attributed to their substantial reduction in coccidia numbers, and *C. perfringens* colonization.

### The combination of usnic acid and tannic acid improves growth performance by increasing the abundance of SCFAs-associated bacteria

Gut microbes contribute to enhanced animal performance by facilitating nutrient absorption and supporting immune function. To examine the impact of usnic acid and its combination with tannic acid on intestinal health in NE-challenged broilers, the intestinal microbiota structure was analyzed using 16S sequencing. These findings revealed that NE treatment did not significantly reduce microbial α diversity, which aligns with the findings of previous studies [56, 57]. However, contrasting studies have reported a reduction in α diversity following NE exposure [50], potentially influenced by factors such as the timing of NE onset and the site of intestinal sample collection [58]. Increased α diversity, reduced AVD values, and enhanced community stability were observed in the group receiving tannic and usnic acids in combination, favoring resistance to external disturbances. In the ileal microbiome, *Lactobacillus* and Lachnospiraceae were prominent in the UTA group. *Lactobacillus* has been shown to enhance intestinal barrier function, modulate immunity, and regulate metabolism [59, 60]. Lachnospiraceae, a significant producer of SCFAs in the intestine [61], contributed to the elevated acetate and butyrate levels observed in the cecum of the UTA group. SCFAs provide energy to intestinal epithelial cells, potentially explaining the higher VH:CD ratios in this group. NE-induced reductions in *Lactobacillus* and Lachnospiraceae abundance [62] were mitigated by the combined administration of UA and TA, indicating their beneficial role in gut microbial regulation. In the cecal microbiome, Lactobacillales abundance decreased following NE treatment but increased significantly in the UTA group. Certain *Lactobacillus* species inhibit the growth of *C. perfringens* [63], with lactic acid production reducing the intestinal pH, thereby suppressing the proliferation of acid-sensitive pathogenic bacteria. Research has reported an increased abundance of *Campylobacterales* in the intestines of mice with dysbiotic gut microbiota [64] and newly weaned piglets [65]. Similarly, the present study revealed a relatively high relative abundance of *Campylobacterales* in the cecum of broiler chickens with NE. Furthermore, the relative abundances of *Butyricicoccus* and *Blautia* were reduced in the intestines of broilers with NE, which is consistent with findings from previous studies [36, 66]. These genera produce SCFAs, which play crucial roles in maintaining intestinal barrier integrity and exerting anti-inflammatory effects [67–69]. Additionally, *Blautia* is a dominant genus in the gut microbiota and has probiotic attributes, including the ability to modulate host health and mitigate metabolic syndrome [70]. The increased relative abundance of these genera following tannic and usnic acid supplementation likely contributed to improved growth performance. Collectively, these results suggest that combined supplementation with tannic and usnic acids effectively mitigates NE-induced dysbiosis and excessive mucosal immune responses, with superior gut microbiome modulation compared with individual treatments.

In conclusion, this study evaluated and confirmed the potential of usnic acid and tannic acid as combined feed additives. Nonetheless, certain limitations persist. The precise mechanisms through which the combination of usnic acid and tannic acid inhibits *C. perfringens* in vitro remain unclear, as do the factors contributing to the more substantial improvement in intestinal health observed in vivo. However, further studies are needed to elucidate these underlying mechanisms.

## Conclusions

Usnic acid and tannic acid induce coccidia apoptosis by reducing the mitochondrial membrane potential. Both usnic acid alone and the combination of usnic acid and tannic acid improve intestinal health by suppressing the growth of coccidia and *C. perfringens*, increasing the abundance of beneficial SCFAs-producing bacteria, and alleviating intestinal hyperinflammation and NE-induced permeability. These combined effects contribute to improved growth performance in broilers. Compared with usnic acid, the combination of usnic acid and tannic acid has a stronger inhibitory effect on *C. perfringens* proliferation, significantly increasing the concentrations of SCFAs in the cecum and leading to more significant improvements in the gut microbiota composition and growth performance. Thus, the combined use of usnic acid and tannic acid is more effective than the use of usnic acid alone.

Given the promising results observed in this study, future studies should further explore the optimal dosages and synergistic mechanisms of usnic acid and tannic acid in controlling NE under commercial production conditions. From a practical perspective, incorporating these natural compounds into antibiotic-free poultry diets may offer a sustainable strategy to improve gut health and growth performance while mitigating the risk of antimicrobial resistance.

## Data Availability

All data generated or analyzed during this study are available from the corresponding author upon request. Datasets supporting the conclusions of this study are included in this article. The 16S gene sequencing data can be obtained from the following website: http://www.ncbi.nlm.nih.gov/bioproject/1197183 and http://www.ncbi.nlm.nih.gov/bioproject/1197104; the accession number is PRJNA1197183 and PRJNA1197104.

## Abbreviations

DAO: Diamine oxidase
D-LA: D-Lactic acid
ELISA: Enzyme-linked immunosorbent assay
FCR: The ratio of feed intake and body weight gain
IL-6: Interleukin-6
IL-10: Interleukin-10
NE: Necrotic enteritis
PDGF: Platelet-derived growth factor
SCFA: Short-chain fatty acid
sIgA: Secretory immunoglobulin A
TGF-β: Transforming growth factor-beta
TNF-α: Tumor necrosis factor-alpha
VEGF: Vascular endothelial growth factor
VH/CD: The ratio of villus height to crypt depth
